# 血小板输注无效在慢性粒-单核细胞白血病患者异基因造血干细胞移植后的发生率及临床意义

**DOI:** 10.3760/cma.j.issn.0253-2727.2022.09.005

**Published:** 2022-09

**Authors:** 晨 赵, 晓甦 赵, 昱 王, 晨华 闫, 兰平 许, 晓辉 张, 开彦 刘, 晓军 黄, 于谦 孙

**Affiliations:** 北京大学人民医院，北京大学血液病研究所，国家血液系统疾病临床医学研究中心，造血干细胞移植北京市重点实验室，北京 100044 Peking University People's Hospital, Peking University Institute of Hematology, National Clinical Research Center for Hematologic Disease, Beijing Key Laboratory of Hematopoietic Stem Cell Transplantation, Beijing 100044, China

**Keywords:** 慢性粒单核细胞白血病, 异基因造血干细胞移植, 血小板输注无效, Chronic myelomonocytic leukemia, allogeneic hematopoietic stem cell transplantation, Platelet transfusion refractoriness

## Abstract

**目的:**

分析血小板输注无效在慢性粒-单核细胞白血病（CMML）患者异基因造血干细胞移植（allo-HSCT）后的发生率及其临床意义。

**方法:**

回顾性分析2004年至2021年在北京大学血液病研究所接受allo-HSCT后的55例CMML患者，根据移植后30 d内是否发生血小板输注无效分为血小板输注无效组和血小板输注有效组，比较两组造血重建、移植相关并发症及移植后1年生存情况。

**结果:**

①55例CMML患者中，男28例（50.9％），女27例（49.1％），中位年龄为44（12～63）岁。②14例（25.5％）患者移植后发生血小板输注无效，41例（74.5％）患者血小板输注有效。③血小板输注无效组血小板植入率明显低于血小板输注有效组（28.6％对100％，*χ*^2^＝26.835，*P*<0.001），血小板植入的中位时间分别为67（33～144）d、21（9～157）d（*χ*^2^＝2.696，*P*＝0.010）。血小板输注无效组、有效组急性移植物抗宿主病（aGVHD）和慢性移植物抗宿主病（cGVHD）发生率差异无统计学意义（*χ*^2^＝3.399，*P*＝0.183；*χ*^2^＝1.573，*P*＝0.455）。④血小板输注无效组、有效组移植后1年总生存（OS）率分别为（35.4±13.9）％、（75.1±7.8）％（*χ*^2^＝4.366，*P*＝0.037），无白血病生存（LFS）率分别为（28.1±13.3）％、（65.3±8.2）％（*χ*^2^＝3.226，*P*＝0.072），移植相关死亡率（TRM）分别为（48.2±2.4）％、（9.0±0.25）％（*χ*^2^＝4.747，*P*＝0.009）。⑤血小板输注无效组移植后出血事件发生率高于血小板输注有效组［35.7％（5/14）对4.9％（2/41），*χ*^2^＝6.934，*P*＝0.009］。

**结论:**

CMML患者allo-HSCT后血小板输注无效发生率为25.5％，显著影响血小板植入率和植入速度，且移植后出血事件发生率增加、OS率降低、TRM增高。

## Abstract (en)

**Objective:**

To retrospectively analyze the incidence and clinical significance of platelet transfusion refractoriness (PTR) after allogeneic hematopoietic stem cell transplantation (allo-HSCT) in patients with chronic myelomonocytic leukemia (CMML).

**Methods:**

A cohort of 55 CMML patients received allo-HSCT at Peking University Institute of Hematology during 2004–2021 were retrospectively assessed. The incidence of PTR within 30 days after allo-HSCT was retrospectively analyzed, and the impact on clinical outcomes and bleeding event were compared between patients with platelet transfusion refractoriness (PTR) or effective platelet transfusion (EPT).

**Results:**

The incidence of PTR after allo-HSCT in CMML patients was 25.5％ (14/55). PTR patients had a lower rate of platelet engraftment than EPT patients (28.6％ *vs* 100％), and the median time of engraftment was 67 (33–144) days and 21 (9–157) days respectively (*P*<0.010). There was no significant difference between two groups in acute graft-versus-host disease (aGVHD) and chronic graft-versus-host disease (cGVHD) (*P*＝0.183, *P*＝0.455). After following-up a median of 684 (24–3978) days, the 1-year overall survival (OS) and 1-year leukemia free survival (LFS) in PTR and EPT patients were (35.4±13.9)％ *vs* (75.1±7.8)％ (*P*＝0.037) and (28.1±13.3)％ *vs* (65.3±8.2)％ (*P*＝0.072), respectively. The transplant-related mortality (TRM) were (48.2±2.4)％ and (9.0±0.25)％, respectively (*P*＝0.009). Bleeding events occurred in five patients (35.7％) of PTR and 2 patients (4.9％) of EPT (*P*＝0.009).

**Conclusion:**

In CMML patients with allo-HSCT, the incidence of PTR is 25.5％, which was associated with delayed platelet engraftment, increased bleeding events, inferior OS and increased TRM.

慢性粒-单核细胞白血病（CMML）是一类同时具有骨髓增生异常和骨髓增殖性特征的单克隆骨髓造血异常的恶性肿瘤。CMML的年发病率约为4/10万，预后较差，中位生存时间为12～50个月，近15％～30％的CMML患者转化为白血病[1]–[2]。除了羟基脲、去甲基化药物、细胞毒药物化疗等传统疗法，异基因造血干细胞移植（allo-HSCT）也是目前CMML的重要治疗手段，移植后3年的总生存（OS）率为20％～50％[3]–[6]。

CMML患者allo-HSCT后移植相关死亡率（TRM）为30％～40％，甚至高达52％[7]。在本中心既往报道的19例CMML患者中，2例在移植早期死于颅内出血，可能与持续血小板输注无效（PTR）有关[6]。既往本中心回顾性分析显示allo-HSCT后血小板重建延迟显著增加出血性事件发生率[8]，并显著增加TRM并降低移植后生存率[9]。在获得血小板重建之前，血小板输注是重要的支持治疗手段，可有效预防或治疗出血性事件。然而，部分患者输注血小板后出现血小板输注无效（输注血小板后血小板计数未达到预期水平）。与其他血液恶性疾病相比，CMML患者在移植前即有约40％的患者因血小板减少而反复输血[10]–[11]，移植后血小板植入相对较慢[6]，此外由于其容易合并存在HLA抗体[12]、脾脏肿大[13]等原因，血小板输注无效的发生风险可能更高。本研究对接受allo-HSCT的55例CMML患者进行回顾性分析，旨在探讨CMML患者allo-HSCT后血小板输注无效的发生率及其临床意义。

## 病例与方法

一、病例

本研究纳入2004年1月至2021年1月期间在北京大学血液病研究所诊断为CMML并接受allo-HSCT的55例患者。CMML诊断标准参考2016年WHO诊断标准[14]。根据WHO诊断标准将CMML分为3期：CMML-0：外周血原始细胞<2％，骨髓原始细胞<5％；CMML-1：外周血原始细胞2％～4％和（或）骨髓原始细胞5％～9％；CMML-2：外周血原始细胞5％～19％，骨髓10％～19％和（或）有Auer小体存在。MDAPS评分标准[15]：HGB<120 g/L，淋巴细胞计数>2.5×10^9^/L，外周血原始粒细胞>0％和骨髓原始细胞>10％，根据每项1分，分为低危（0～1分）、中危-1（2分）、中危-2（3分）和高危（4分）。CPSS评分标准[16]：WHO分型，FAB分型，红细胞输注依赖（4个月内每8周至少输注1次红细胞），以及细胞遗传学危险分层。

二、移植及预处理方案

移植方案见文献[17]。

单倍体移植预处理常规方案[18]：阿糖胞苷2 g·m^−2^·d^−1^静脉滴注，−10 d、−9 d；白消安0.8 mg/kg每6 h 1次静脉滴注，−8 d～−6 d；环磷酰胺1.8 g·m^−2^·d^−1^静脉滴注，−5 d、−4 d；司莫司汀250 mg/m^2^口服，−3 d；兔抗人胸腺细胞免疫球蛋白2.5 mg·kg^−1^·d^−1^静脉滴注，−5 d～−2 d。

同胞全相合移植预处理方案：羟基脲800 mg/kg分2次口服，−10 d；阿糖胞苷2 g/m^2^静脉滴注，−9 d；白消安0.8 mg/kg每6 h 1次静脉滴注，−8 d～−6 d；环磷酰胺1.8 g·m^−2^·d^−1^静脉滴注，−5 d、−4 d；司莫司汀250 mg/m^2^口服，−3 d。

三、血小板输注无效的定义

本组病例预防性血小板输注的指证：①PLT<20×10^9^/L；②PLT≥20×10^9^/L但伴有出血倾向。血小板输注后20～24 h复查血小板计数，按照以下公式计算血小板计数恢复率：

$$血小板计数恢复率（％）=\frac{血小板计数增量}{血小板量输注量}\times 血容量（L）$$

血小板恢复率≤20％定义为无效反应。血小板输注无效定义为反复出现（≥2次）血小板输注后患者血小板计数恢复率≤20％[19]–[20]。

四、定义

出血事件分级参考原发免疫性血小板减少症的出血评分系统[21]。中性粒细胞植活：移植后中性粒细胞绝对计数（ANC）≥0.5×10^9^/L连续3 d；血小板植活：移植后血小板计数≥20×10^9^/L连续7 d且脱离血小板输注。急性及慢性移植物抗宿主病（GVHD）诊断标准参照文献[22]。

五、随访

随访资料来自于门诊/住院病历和电话随访。随访截止日期为2021年6月30日。中位随访时间684（24～3978）d。OS时间：移植物末次回输至随访截止或死亡的日期。无白血病生存（LFS）时间：移植物末次回输至复发或死亡的日期，未发生者到随访截止。

六、统计学处理

采用SPSS软件进行数据分析。分类变量采用*χ*^2^检验或fisher精确检验，连续变量采用Student's *t*检验或MannWhitney *U*检验。采用Kaplan-Meier法绘制生存曲线，OS、LFS率的组间比较采用Log-rank检验。采用Cox模型进行单因素和多因素分析，将*P*<0.05的参数纳入Cox多因素分析。复发、移植相关死亡为竞争风险，采用R软件cmprsk包进行竞争风险生存模型的比较。

## 结果

一、患者基线特征

全部55例CMML患者中男28例（50.9％），女27例（49.1％），中位年龄为44（12～63）岁。4例患者在移植前即出现血小板输注无效，10例患者移植后30 d内发生血小板输注无效，中位发生时间为移植后16（7～29）d。根据患者移植后30 d内血小板输注情况分为血小板输注无效组（14例）和血小板输注有效组（41例），两组在年龄、性别、移植前是否接受治疗、移植前血小板输注情况、移植前是否伴有脾脏肿大、移植到诊断的时间、WHO分期、FAB分期、CPSS分层、MDAPS分层、供者类型、供受者性别、供患者血型、单个核细胞和CD34^+^细胞输注量方面差异均无统计学意义（*P*>0.05），详见表1。

**表1:** 异基因造血干细胞移植后血小板输注无效、有效慢性粒-单核细胞白血病（CMML）患者临床特征比较

特征	血小板输注无效组（14例）	血小板输注有效组（41例）	*χ*^2^值	*P*值
中位年龄［岁，*M*（范围）］	43（16～63）	41（12～56）	0.232	0.771
性别［例（男/女）］	7/7	21/20	0.010	0.591
WHO分期［例（％）］			2.974	0.301
CMML-0	4（28.6）	5（12.2）		
CMML-1	5（35.7）	14（34.1）		
CMML-2	5（35.7）	22（53.7）		
FAB分期［例（％）］			0.074	0.510
骨髓增生异常型	6（42.9）	12（29.3）		
骨髓增殖型	8（57.1）	29（70.7）		
移植前治疗［例（％）］			0.262	0.762
有	7（50.0）	18（43.9）		
无	7（50.0）	23（56.1）		
移植前病程［月，*M*（范围）］	9（1～24）	5（1～24）	1.349	0.117
CPSS分层［例（％）］			0.394	0.524
低危	0（0）	0（0）		
中危-1	1（7.1）	1（2.4）		
中危-2	9（64.3）	32（78.0）		
高危	4（28.6）	8（19.5）		
MDAPS分层［例（％）］			2.379	0.417
低危	4（28.6）	6（14.6）		
中危-1	5（37.5）	12（29.3）		
中危-2	4（28.6）	13（31.7）		
高危	1（7.1）	10（24.4）		
移植前血小板输注依赖［例（％）］	3（21.4）	5（12.2）	1.412	0.327
移植前脾大［例（％）］			0.891	0.529
有	4（28.6）	17（41.5）		
无	10（71.4）	24（58.5）		
供者类型［例（％）］			0.002	0.125
单倍体相合	11（78.6）	22（53.7）		
同胞全相合	3（21.4）	19（46.3）		
供者性别［例（男/女）］	8/6	24/17	0.865	0.927
供、患者血型			2.229	0.309
相合	9（64.3）	17（41.5）		
主要不合	3（21.4）	17（41.5）		
次要不合	2（14.3）	7（17.1）		
MNC输注量［×10^8^/kg，*M*（范围）］	7.41（6.70～9.68）	8.21（5.98～12.00）	1.092	0.384
CD34^+^细胞输注量［×10^6^/kg，*M*（范围）］	2.12（1.09～4.98）	4.05（1.85～8.87）	1.617	0.413

注：MNC：单个核细胞

血小板输注无效组14例患者均检测了人组织相容性抗体（HLA）及抗人血小板（HPA）抗体。其中8例（57.1％）患者为HPA抗体或HLA抗体阳性。4例（28.6％）患者移植前伴脾脏肿大。

二、造血重建

血小板输注无效组14例患者中13例获得粒细胞植活（1例因移植后并发症早期死亡），血小板输注有效组41例患者全部获得粒细胞植活，中位植入时间分别为15（11～25）d、12（10～22）d（*χ*^2^＝1.697，*P*＝0.628）。血小板输注无效组、有效组血小板植入率分别28.6％（4/14）、100％（41/41）（*χ*^2^＝26.835，*P*<0.001），中位植入时间分别为67（33～144）d、21（9～157）d（*χ*^2^＝2.696，*P*＝0.010）。

三、GVHD发生情况

血小板输注无效组9例（64.3％）患者发生aGVHD，中位发生时间为31（9～83）d，其中5例患者为Ⅰ/Ⅱ度aGVHD，4例为Ⅲ/Ⅳ度aGVHD。血小板输注有效组18例（43.9％）发生aGVHD，中位发生时间为45（12～93）d，其中14例为Ⅰ/Ⅱ度aGVHD，4例为Ⅲ/Ⅳ度aGVHD，两组aGVHD发生率差异无统计学意义（*χ*^2^＝3.399，*P*＝0.183）。血小板输注无效组2例患者发生cGVHD（其中广泛型1例），血小板输注有效组12例患者发生cGVHD（其中广泛型3例），两组cGVHD发生率差异无统计学意义（*χ*^2^＝1.573，*P*＝0.455）。

四、移植后1年生存率

至随访截止，血小板输注无效组7例患者死亡，移植后1年OS率为（35.4±13.9）％，血小板输注有效组15例死亡，移植后1年OS率为（75.1±7.8）％（*χ*^2^＝4.366，*P*＝0.037）（图1A）。两组1年LFS率分别为（28.1±13.3）％、（65.3±8.2）％（*χ*^2^＝3.226，*P*＝0.072）（图1B）。

**图1:**
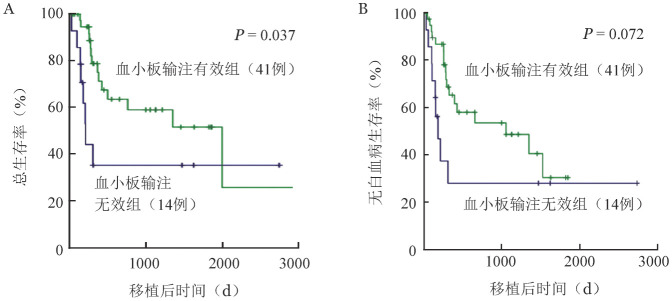
异基因造血干细胞移植后血小板输注无效、有效慢性粒-单核细胞白血病患者移植后总生存曲线（A）和无白血病生存曲线（B）

五、复发

血小板输注无效组3例患者复发，中位复发时间为152（52～305）d，移植后1年累积复发率为（23.6±1.7）％，血小板输注有效组13例患者复发，中位发生时间为405（41～1525）d，移植后1年累积复发率为（25.7±0.6）％，两组比较差异无统计学意义（*χ*^2^＝0.714，*P*＝0.390）。血小板输注无效组有1例患者尚未接受治疗而死亡，1例患者接受化疗+供者淋巴细胞输注后存活，1例患者接受化疗+供者淋巴细胞输注无效而死亡。

六、移植相关死亡

血小板输注无效组7例患者死亡，其中5例为移植相关死亡，其中2例血小板植入失败并死于脑出血，1例死于弥漫性肺泡出血，2例因合并重症感染死亡，移植后1年TRM为（48.2±2.4）％。血小板输注有效组15例死亡，其中6例为移植相关死亡，其中只有1例因脑出血死亡，1例因出现闭塞性细支气管炎治疗无效死亡，4例因重症感染死亡，1年TRM为（9.0±0.25）％。血小板输注无效组TRM高于血小板输注有效组（*χ*^2^＝4.747，*P*＝0.009）。

七、出血事件

血小板输注无效组5例患者（35.7％）发生出血事件（均为重度出血），其中脑出血2例、弥漫性肺泡出血2例、上消化道出血1例，3例有血小板抗体和HLA抗体，3例因严重出血事件死亡，其中2例有血小板抗体和HLA抗体存在。血小板输注有效组2例（4.9％）发生出血事件，脑出血、上消化道出血各1例。血小板输注无效组出血事件发生率高于血小板输注无效组（*χ*^2^＝6.934，*P*＝0.009）。

## 讨论

以往文献报道allo-HSCT后血小板输注无效的发生率为7％～55％[20],[23]–[24]，本组CMML患者allo-HSCT后血小板输注无效的发生率为25.5％。目前尚无CMML患者与急性白血病等其他血液系统疾病移植后血小板输注无效发生率的比较数据。但CMML患者具有与急性白血病不同的疾病特点，可能是影响血小板输注无效发生率差异的原因：①输血依赖需求较高：既往一项分析提示骨髓增生异常综合征（MDS）患者移植后更容易发生血小板输注无效，推测可能与MDS患者输血史有关[25]；②与急性白血病相比，CMML患者移植前后对于血小板输注的需求更大，移植前血小板减少症在CMML患者中的发生率约40％[26]，频繁的血小板输注增加了产生血小板抗体的风险；③约30％的CMML患者移植时伴有脾脏肿大，而脾肿大可能也是CMML患者发生血小板输注无效的风险之一[13],[27]。本组55例CMML患者移植后血小板输注无效的发生率高达25.5％，需要给予充分重视。

移植后血小板输注无效的主要机制包括免疫性和非免疫性，前者主要是HLA抗体或抗血小板抗体，后者主要是感染、脾肿大等原因[28]。导致CMML患者移植后发生血小板输注无效的机制可能包括：①既往的研究表明，CMML同多种自身免疫相关疾病有关，发生率约为10％[29]，其发生原因可能与单核细胞功能障碍导致持续性免疫反应，从而增加自身抗体的生成有关。②血小板减少是CMML患者常见的并发症，约40％的患者存在血小板输注依赖，而频繁输血依赖是产生血小板抗体的重要危险因素之一。③HLA抗体是血小板输注无效的主要机制之一，而MDS/CMML患者中存在HLA抗体的风险较高，既往本中心的报道显示MDS患者中出现HLA抗体的风险显著高于非MDS患者[12],[30]。④由于肿大的脾脏常常滞留循环中的血小板，导致患者合并非免疫性血小板输注无效，脾脏肿大也是导致血小板输注无效的原因之一[13]；CMML患者在移植前30％存在脾脏肿大，而且移植后短期脾脏肿大并不能快速缓解，且脾脏肿大患者伴有显著的移植后血小板植入延迟[27]，因此脾脏肿大可能是CMML患者血小板输注无效的发生机制之一。

既往研究显示，恶性血液病患者allo-HSCT后血小板输注无效的危险因素包括CD34^+^细胞输注量较少、抗生素大量应用、存在HLA抗体及减低剂量预处理方案[31]，Tanoue等[23]在一项单中心回顾性研究中纳入185例接受脐带血移植患者，结果显示移植后血小板输注无效的危险因素包括有怀孕史的女性、男性、存在HLA抗体、较低的脐带血单核细胞数、体温38 °C，C反应蛋白>10 mg/L、巨细胞病毒血症、应用膦甲酸钠、应用两性霉素B。本研究未能发现与血小板输注无效发生相关的因素，可能与本研究样本数量较小有关。

本研究结果显示，移植后血小板输注无效的发生可导致血小板植入失败和植入时间延迟，但对中性粒细胞植入无影响，Tanoue等[23]的研究结果也显示血小板输注无效影响血小板植入。本组CMML患者allo-HSCT后血小板输注无效对移植后生存和移植相关死亡有明显影响，与文献[20], [23]结果相同。此外，血小板输注无效组出血事件明显增加，且均为重度出血事件，严重影响移植预后，因此关注CMML患者移植后血小板输注无效的发生，提前进行干预，对预后有重要意义。

研究表明，血小板生成素（TPO）受体激动剂可促进移植后血小板输注无效患者血小板恢复[10],[26]，但目前缺乏治疗相关的大型研究数据。随着新型药物以及治疗手段的不断提高，其疗效评估仍需大规模研究进一步证实，糖皮质激素、血小板受体激动剂、免疫球蛋白等治疗可能有效[10]。加强移植前血小板减少症的管理及治疗，对移植后血小板输注无效甚至植入不良的发生发展可能对减少CMML移植后致命性出血事件、改进CMML移植预后有一定意义。

总之，本研究提示CMML患者allo-HSCT后血小板输注无效发生率较高，伴随血小板植入延迟、出血事件增加，使移植后1年OS率降低。本研究为单中心回顾性研究，且样本量较少，上述结论仍需大规模、多中心研究进一步证实。
